# The effects of secondary iron overload and iron chelation on a radiation-induced acute myeloid leukemia mouse model

**DOI:** 10.1186/s12885-021-08259-9

**Published:** 2021-05-06

**Authors:** Lap Shu Alan Chan, Lilly ChunHong Gu, Richard A. Wells

**Affiliations:** 1 Biological Sciences, Sunnybrook Research Institute Sunnybrook Health Sciences Centre, Toronto, ON Canada; 2 Department of Medical Biophysics, University of Toronto, 101 College St Suite 15-701, Toronto, Ontario M5G1L7 Canada; 3 Odette Cancer Centre, Sunnybrook Health Sciences Centre, Toronto, ON Canada; 4 Current Address: Office of the Chief Coroner for Ontario, 25 Morton Shulman Avenue, Toronto, ON Canada

**Keywords:** Secondary iron overload, Iron chelation, Radiation induced acute myeloid leukemia, Leukemogenesis, Myelodysplastic syndrome

## Abstract

**Background:**

Patients with myelodysplastic syndrome (MDS) require chronic red blood cell (RBC) transfusion due to anemia. Multiple RBC transfusions cause secondary iron overload and subsequent excessive generation of reactive oxygen species (ROS), which leads to mutations, cell death, organ failure, and inferior disease outcomes. We hypothesize that iron loading promotes AML development by increasing oxidative stress and disrupting important signaling pathways in the bone marrow cells (BMCs). Conversely, iron chelation therapy (ICT) may reduce AML risk by lowering iron burden in the iron-loaded animals.

**Methods:**

We utilized a radiation-induced acute myeloid leukemia (RI-AML) animal model. Iron overload was introduced via intraperitoneal injection of iron dextran, and iron chelation via oral gavage of deferasirox. A total of 86 irradiated B6D2F1 mice with various levels of iron burden were monitored for leukemia development over a period of 70 weeks. The Kaplan-Meier estimator was utilized to assess AML free survival. In addition, a second cohort of 30 mice was assigned for early analysis at 5 and 7 months post-irradiation. The BMCs of the early cohort were assessed for alterations of signaling pathways, DNA damage response and gene expression. Statistical significance was established using Student’s t-test or ANOVA.

**Results:**

Iron loading in irradiated B6D2F1 mice accelerated RI-AML development. However, there was a progressive decrease in AML risk for irradiated mice with increase in iron burden from 7.5 to 15 to 30 mg. In addition, ICT decreased AML incidence in the 7.5 mg iron-loaded irradiated mice, while AML onset was earlier for the 30 mg iron-loaded irradiated mice that received ICT. Furthermore, analysis of BMCs from irradiated mice at earlier intervals revealed accelerated dysregulation of signaling pathways upon iron loading, while ICT partially mitigated the effects.

**Conclusions:**

We concluded that iron is a promoter of leukemogenesis in vivo up to a peak iron dose, but further iron loading decreases AML risk by increasing cell death. ICT can partially mitigate the adverse effects of iron overload, and to maximize its benefit this intervention should be undertaken prior to the development of extreme iron overload.

**Supplementary Information:**

The online version contains supplementary material available at 10.1186/s12885-021-08259-9.

## Background

Myelodysplastic syndrome (MDS) patients who are chronically anemic require sustained red blood cell (RBC) transfusion, which inevitably leads to secondary hemochromatosis with significant pathophysiological consequences. To mitigate the risk of iron-related morbidity and premature mortality, iron chelation therapy (ICT) is recommended in transfusion dependent iron-overloaded patients with lower risk MDS, even though the evidence that ICT is effective in influencing these outcomes is less extensive than it is for patients with thalassemia major [1].

Retrospective reviews of registry data suggested that iron overload in MDS may promote the development of acute myeloid leukemia (AML) [2]. Although an excess incidence of AML is not seen in thalassemia major, such an association in MDS may nonetheless be plausible. Hereditary hemochromatosis is associated with several forms of human carcinomas in liver, lung and colon [3], hence establishing the principle that iron can promote cancer development. In addition, it must be borne in mind that MDS – a clonal myeloid disorder that is intrinsically linked to AML development and that is characterized by genomic instability – can be expected to be more susceptible than thalassemia major to an AML-promoting effect.

Proof that iron overload promotes the progression of MDS to AML would have a profound effect on the aims and breadth of ICT in this disease, and could have a positive impact in both low and high risk MDS. We utilized a radiation-induced AML (RI-AML) B6D2F1 mouse model to investigate the hypothesis that extrinsic iron overload can promote AML development.

## Methods

### Animals

B6D2F1 mice were obtained from and Charles River Canada (St. Constant, QC, Canada). Upon arrival, all mice were randomized and housed in unisex groups of 5 or less per cage in a temperature and humidity-controlled room maintained on a 12:12 h light/dark cycle in a pathogen-free facility at the Sunnybrook Research Institute.

### Treatments

All chemicals were purchased from Sigma-Aldrich (St. Louis, MO, USA) unless stated otherwise. For radiation treatment, nine-week-old male B6D2F1 mice were subjected to non-lethal total-body irradiation at 300 cGy delivered in a Cs-137 small animal irradiator. Three hours after irradiation, the mice were inoculated with 0.5 mg dexamethasone sodium phosphate (Omega Laboratories, Montreal, QC, Canada) by subcutaneous injection. The use of dexamethasone has been reported to increase leukemia incidence by up to 50% in irradiated SJL/J mice [4]. Mice began to receive iron or sham treatment at 2 weeks after irradiation. Iron dextran (1 mg iron equivalent) or the corresponding dosage of dextran (from *Leuconostoc* spp., M_r_ ~ 6000) was delivered by intraperitoneal injection for 5 days per week until the desired iron burden was reached. Iron burden is defined as the total amount of excess iron in the body after iron loading and/or ICT. ICT was initiated after the end of iron loading. Deferasirox (Novartis, Dorval, QC, Canada) was suspended in 0.5% hydroxypropylcellulose (a gift from Nippon Soda Co. Ltd., Tokyo, Japan) and administered by oral gavage at 10 or 40 mg/kg/day for 7 days a week over 4 or 8 weeks.

The main cohort (Fig. S1) consisted of 8 treatment groups with varying iron (mg)/ICT (mg/kg/day) dosage (total number = 86): 0/0 (control, *N* = 13), 7.5/0 (N = 13), 15/0 (*N* = 10), 30/0 (N = 10), 7.5/10 (N = 10), 7.5/40 (N = 10), 30/10 (N = 10), and 30/40 (N = 10). The individual mouse was considered to be an experimental unit. The sample size calculation was based on 30% RI-AML incident rate [4], with 30% margin of error at 95% confidence level – a smaller samples size was selected in order to screen a wider range of iron burden and iron chelation dosage. In addition, a separate cohort of 30 animals were assigned for early analysis at 5 and 7 months post-irradiation (Fig. S1): 0/0, 5/0, 5/40 for 5 months (*N* = 5 per group); 0/0, 7.5/0, 7.5/40 for 7 months (*N* = 5 per group).

### Monitoring and analysis

The body weight of all irradiated B6D2F1 mice was measured weekly for the 70 weeks post-irradiation observation period. Overt leukemia was suspected when the subject lost 20% of its body weight, showed signs of illness, and presented leukemic blasts in its tail vein peripheral blood (PB) smear. Diagnosis of AML was made based on the Bethesda proposals for classification of nonlymphoid hematopoietic neoplasms in mice [5]. The mice were sacrificed using asphyxiation with carbon dioxide followed by cervical dislocation according to the following criteria: 1) became ill (20% weight lost, lack of activity, hunched posture, etc), 2) assigned for early analysis, 3) after the 70 weeks observation period. Tissue samples, including PB, hind limb bones, spleen, liver, and heart, were collected and analyzed. PB count was measured by the Sunnybrook Health Science Centre Hematology Laboratory Service. PB morphology was evaluated by May-Grünwald-Giemsa staining of air-dried smears. At least one of the hind limb bones and other harvested organs were fixed for 24 h in neutral buffered 10% formalin solution followed by decalcification of the bones, paraffin embedding, slicing, and staining with hematoxylin and eosin by the Sunnybrook Research Institute Histology Core Facility. Iron accumulation was also confirmed in the organs of the iron-loaded mice using Prussian Blue staining (not shown).

### Bone marrow cells (BMCs) processing and analysis

BMCs for each mouse were obtained from the remaining hind limb bones by flushing the medullary cavity with PBS containing 2% fetal bovine serum (FBS, Life Technologies, Burlington, ON, Canada). Mature RBC from the BMCs were lysed using ACK lysing buffer (Life Technologies). The BMCs were then washed and resuspended in PBS with 2% FBS for subsequent flow cytometry and DNA/RNA/protein analysis (See the supplementary methods for further details). An aliquot of BMCs was also prepared for cytospin and stained with May-Grünwald-Giemsa stains for the evaluation of cell morphology.

### Figures and data analysis

All figures and statistical analysis were prepared by GraphPad Prism 5 (GraphPad Software, Inc., La Jolla, CA, USA) or Microsoft Excel. Data were presented as mean ± SD. Statistical significance was determined by Student’s t-test or analysis of variance (ANOVA) and set at *P* < 0.05. Post-hoc analysis of significant ANOVA results was performed using the Tukey’s method. Homogeneity of variances was assessed by F-test or Bartlett’s test. AML free survival (AFS) between different treatment groups was plotted on Kaplan-Meier (KM) survival curves and analyzed by the Mantel-Cox test or log-rank test for trend. All statistical tests were two sided. Clustergram for RT^2^ Profiler PCR Arrays was created by the web-based algorithm provided by QIAGEN.

## Results

### Effects of iron and ICT on radiation-induced AML

**Fig. 1:**
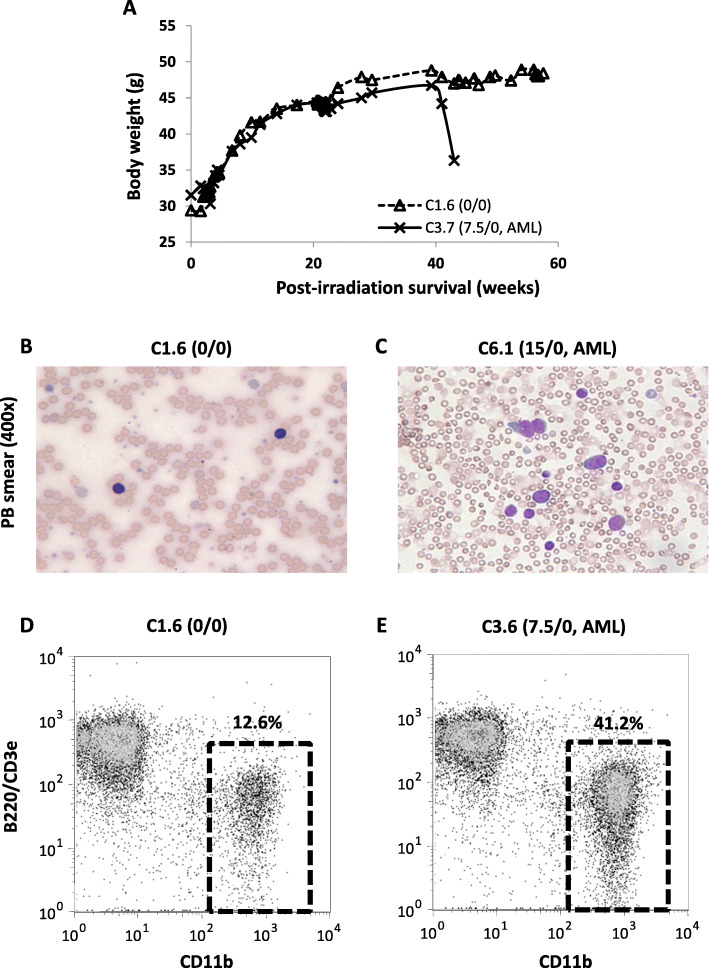
Manifestation of AML in irradiated mice. **a** Representative weight change over time for a non-leukemic mouse (C1.6) and a leukemic mouse (C3.7). Representative May-Grünwald-Giemsa staining of peripheral blood (PB) smear for mouse C1.6 (**b**) and C6.1 (**c**). Representative flow cytometry analysis of myeloid vs. lymphoid population in PB for mouse C1.6 (**d**) and C3.6 (**e**)

During the 70 weeks observation period, 28 (32.6%) of the 86 mice were found dead or needed to be sacrificed due to illness (Table 1). Among these mice, 15 were diagnosed with AML, while the other 13 died from other causes ranging from preputial abscess to tumors at various locations (Table 2). Since irradiation in mice is specifically associated with AML [4], we decided to censor other causes of death and focus our analysis on AML. Although beyond the scope of this study, it is reasonable to speculate that tumor development at other sites is associated with iron toxicity. Manifestation of AML was marked by severe weight drop of at least 20% and the presence of blasts in PB (Fig. 1 a-c). In mice with leukemia, the size of the myeloid compartment in the PB, based on CD11b^+^ population, was also expanded in the PB in comparison to B220^+^CD3e^+^ lymphoid population (Fig. 1 d, e). The condition was fatal within 4 weeks after the weight drop. Other manifestations of AML include hepatomegaly or splenomegaly (Table 2), blast infiltration into organs (Fig. S2), homogenous BMCs with high proportion of immature cells (Fig. S3), expansion of immature (Lin^−^CD45^+^ or Lin^−^CD45^low/−^) hematopoietic populations (Fig. S4), and the presence of CD11b^−^Gr-1^+^ population in the BMCs (Fig. S5).

**Table 1:** Characteristics of the B6D2F1 mice in the main cohort (C1.X to C16.X)

Treatment (iron/ICT)	0/0	7.5/0	7.5/10	7.5/40	15/0	30/0	30/10	30/40
	C1.XC2.X	C3.XC4.X	C9.XC10.X	C11.XC12.X	C5.XC6.X	C7.XC8.X	C13.XC14.X	C15.XC16.X
N	13	13	10	10	10	10	10	10
Total iron burden (mg)	0	7.5	7.5	7.5	15	30	30	30
Deferasirox (mg/kg/d)	0	0	10	40	0	0	10	40
Mean initial body weight (g)	27.12 ± 1.57	28.21 ± 2.1	32.93 ± 2.85	30.51 ± 1.79	27.68 ± 3.32	27.32 ± 1.78	31.19 ± 1.48	31.69 ± 3.11
Mean maximum body weight (g)	52.35 ± 3.37	50.48 ± 7.05	56.37 ± 4.64	50.38 ± 4.13	52.56 ± 6.05	55.91 ± 10.89*	51.95 ± 5.27	56.73 ± 7.52
Mortality due to AML	0 (0%)	5 (38%)	1 (10%)	3 (30%)	2 (20%)	1 (10%)	3 (30%)	0 (0%)
Total mortality	2 (15%)	7 (54%)	3 (30%)	4 (40%)	3 (30%)	3 (30%)	3 (30%)	4 (40%)

* F-test compared to control *P* < 0.0005

**Table 2:** Cause of death (AML or non-AML) in the main cohort (C1.X to C16.X)

Label	Iron/ICT^**a**^	AFS/OS (weeks)^**b**^	Final body weight (g)	Final liver weight (g)	Final spleen weight (mg)	Diagnosis^**c**^
*AML*						
C3.3	7.5/0	35.8/37.1	33.5	2.53	260	Monocytic leukemia
C3.6	7.5/0	54.0/57.0	45.0	–	–	Myeloid leukemia^d^
C3.7	7.5/0	41.0/43.0	36.3	–	–	Myeloid leukemia^d^
C4.1	7.5/0	58.7/60.0	47.1	3.83	1085	Monocytic leukemia
C4.5	7.5/0	25.4/27.7	29.0	2.22	367	Monocytic leukemia
C10.1	7.5/10	31.3/35.0	31.6	7.29	1560	Monocytic leukemia
C11.4	7.5/40	37.3/38.3	34.1	3.64	1609	Monocytic leukemia
C11.5	7.5/40	63.1/69.0	40.7	2.06	86	Myeloid leukemia^e^
C12.5	7.5/40	36.3/41.6	36.4	2.01	930	Myelomoncytic leukemia
C6.1	15/0	55.7/57.0	42.9	3.94	789	Monocytic leukemia
C6.5	15/0	52.7/54.0	52.2	4.01	579	Myeloid leukemia^d^
C7.4	30/0	67.1/67.1	58.9	3.42	548	Myelomoncytic leukemia
C13.3	30/10	42.1/45.0	36.9	3.80	742	Myeloid leukemia^d^
C13.4	30/10	33.3/36.4	33.1	6.01	1170	Monocytic leukemia
C14.2	30/10	55.6/55.6	41.3	3.32	293	Myeloid leukemia^d^
*Non-AML (censored)*						
C1.4	0/0	-−−/56.6	43.9	2.04	97	Other
C2.4	0/0	-−−/50.0	49.9	2.12	1380	Metastasized tumor
C4.4	7.5/0	-−−/16.7	37.2	1.46	63	Other
C10.2	7.5/10	-−−/69.3	51.8	1.78	80	Other
C10.5	7.5/10	-−−/68.6	45.5	6.30	175	Liver tumor
C12.1	7.5/40	-−−/69.1	35.6	1.13	140	Lung tumor
C5.1	15/0	-−−/67.1	52.0	2.88	187	Abdominal tumor
C7.3	30/0	-−−/52.6	37.3	–	–	Other
C8.5	30/0	-−−/39.6	28.4	2.08	87	Other
C15.1	30/40	-−−/54.6	38.9	2.71	421	Lymphoid neoplasm
C15.2	30/40	-−−/31.6	29.2	1.88	89	Liver tumor
C15.4	30/40	-−−/44.0	60.8	3.87	205	Bone tumor
C16.2	30/40	-−−/67.4	58.0	3.40	389	Abdominal tumor

^a^ Iron burden (mg) / ICT by deferasirox (mg/kg/d)

^b^ AML-free survival / overall survival (post-irradiation, weeks)

^c^ Diagnosis of AML was made based on the Bethesda proposals for classification of nonlymphoid hematopoietic neoplasms in mice

^d^ AML subtype not determined

^e^ Myeloid leukemia with maturation

**Fig. 2:**
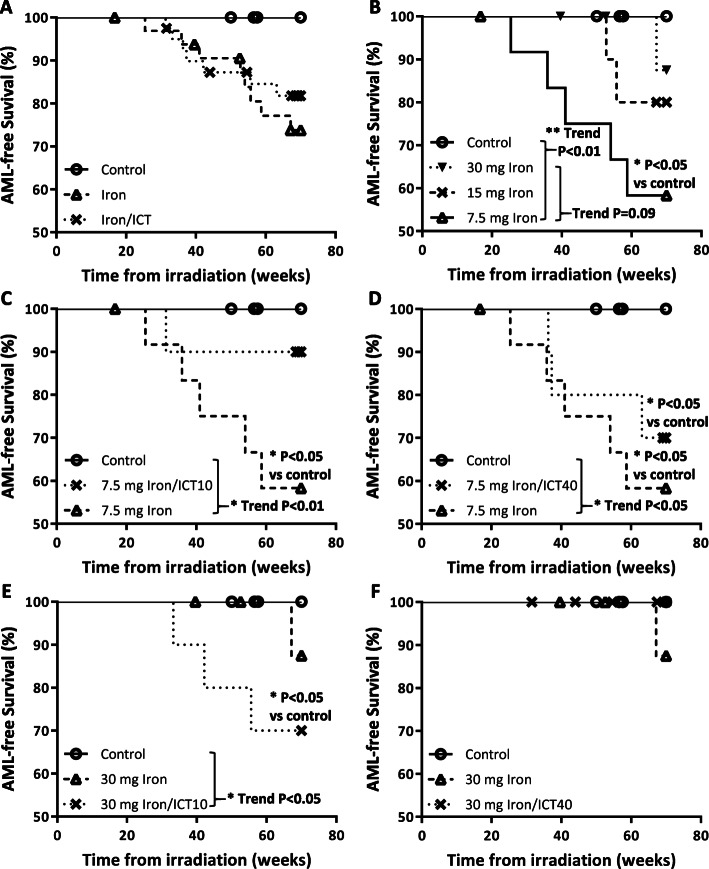
Kaplan-Meier (KM) AML-free survival curves. **a** Control group (Iron/ICT: 0/0) versus aggregated iron injected groups (7.5/0, 15/0, 30/0) and aggregated iron/ICT treatment groups (7.5/10, 7.5/40, 30/10 and 30/40). **b** Control (0/0) and iron injected groups (7.5/0, 15/0, 30/0). **c** Low iron and low ICT (0/0, 7.5/0, 7.5/10). **d** Low iron and high ICT (0/0, 7.5/0, 7.5/40). **e** High iron and low ICT (0/0, 30/0, 30/10). **d** High iron and high ICT (0/0, 30/0, 30/40). Statistical significance of treatment vs control was established using Mantel-Cox test. Trend analysis was established using Logrank test for trend according to order listed in the legend of each chart

Based on the Bethesda proposals for classification of nonlymphoid hematopoietic neoplasms [5], 7 mice were diagnosed with monocytic leukemia characterized by the presence of a monocytic component in the BMCs, 2 mice had myelomoncytic leukemia with both neutrophilic and monocytic components, and one mouse had myeloid leukemia with maturation characterized by the presence of a neutrophilic component (Table 2). The other 5 mice were confirmed to have myeloid leukemia, but the exact subtype could not be determined (Table 2). All of the AML cases during the observation period developed in mice that belonged to the iron-loaded or iron-loaded/ICT groups. Of the control mice that were irradiated but not iron-loaded, two eventually developed AML after the end of the observation period. Within the observation period, the earliest AML onset was at 25 weeks after irradiation and the latest was at 67 weeks. AFS in iron-loaded and iron-loaded/ICT mice were 74 and 82%, respectively, and both were not significantly different when compared with the control mice (*P* = 0.06 and *P* = 0.12, respectively) or with each other (*P* = 0.49 Fig. 2a). Among the iron-loaded mice, the highest rate of AML was observed in the group receiving 7.5 mg iron with AFS at 58% (*P* < 0.05, HR 9.29 vs controls, Fig. 2b). Surprisingly, there appeared to be an inverse relationship between iron dose and AML. The AFS and earliest AML onset in the 7.5 mg, 15 mg, and 30 mg iron-loaded groups were 58%/25 weeks, 80%/52 weeks, and 88%/67 weeks, respectively (logrank test for trend *P* = 0.09).

Iron chelation by deferasirox at 10 mg/kg/day improved AFS in the 7.5 mg iron-loaded group (90% for 7.5/10 vs 58% for 7.5/0, Fig. 2c). In addition, a significant trend in AFS was observed when the groups were arranged according to the iron burden, ranking lowest-to-highest from control, 7.5/10, to 7.5/0 (trend *P* < 0.01). The mice that receive high-dose deferasirox at 40 mg/kg/day had inferior AFS than their low-dose counterpart (AFS of 70% for 7.5/40, Fig. 2d). Nevertheless, the AFS of the 7.5/40 group was better than the 7.5/0 group and the significant trend persisted in accordance with the iron burden (control to 7.5/40 to 7.5/0, trend *P* < 0.05). Low-dose ICT had the opposite effect on the 30 mg iron-loaded group, in which a more rapid AML onset with inferior AFS was observed after deferasirox treatment (AFS of 70% for 30/10 vs 87.5% for 30/0, Fig. 2e). Bronze discoloration was observed in the ICT-treated 30 mg iron-loaded mice (not shown), suggesting that our ICT protocol did not result in the complete removal of excess iron. Remarkably, none of the high-iron high-ICT mice had AML (30/40, Fig. 2f), but 40% of them developed various types of tumor (Table 2).

### The role of iron loading in leukemogenesis of RI-AML

To elucidate the mechanism by which iron contributes to radiation-induced leukemogenesis, we examined the irradiated BMCs (iBMCs) from a separate cohort of mice at earlier intervals of 5 and 7 months after irradiation (Fig. S1). We injected the mice with 5 mg iron dextran for the 5 months cohort or 7.5 mg iron dextran for the 7 months cohort, which had the highest incidence rate of AML among the tested iron doses. To assess the effects of ICT, some of the iron-loaded mice had also received oral deferasirox at 40 mg/kg/day. None of the mice from the early cohort developed overt AML. For comparison, we used iBMCs from 5 of the mice in the main cohort that developed AML: C2.5 (0/0), C6.1 (15/0), C7.4 (30/0), C11.4 (7.5/40), and C13.4 (30/10).

**Fig. 3:**
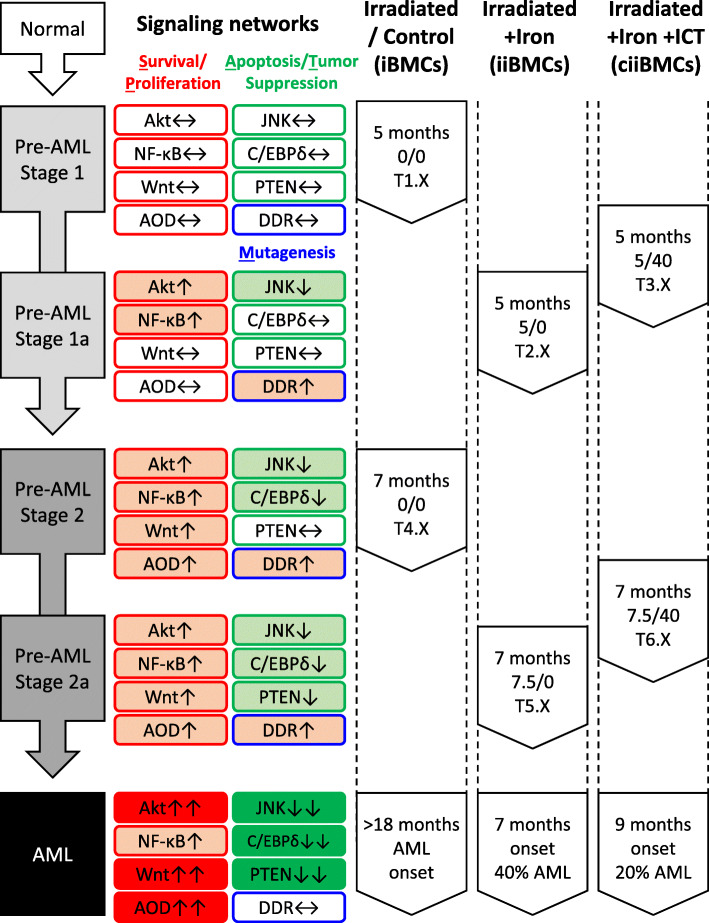
Leukemogenesis, changes of signaling networks, and the influence of iron and ICT. Progressive enhancement (light to dark red fill) and suppression (light to dark green fill) of various signaling networks were listed with their corresponding leukemic stages (normal to pre-AML to AML). Signaling networks were grouped into survival/proliferation (S/P, red outline), apoptosis/tumor suppression (A/T, green outline) and mutagenesis (M, blue outline) based on the combined gene expressions and biomarkers analysis. AOD – antioxidant defenses, DDR – DNA damage response

Assessments of the iBMCs from the earlier cohort alongside the AML group suggested a pattern of progressive molecular changes including alteration of signaling pathways, DNA damage response, and gene expression pattern (see supplementary results). We characterized 3 stages of radiation-induced leukemogenesis in the iBMCs without iron loading from pre-AML stage 1 at 5 months post-irradiation (0/0), to pre-AML stage 2 at 7 months post-irradiation (0/0), to eventual AML beyond 18 months (Fig. 3). There were progressive alterations including activation of Akt, NF-κB, Wnt, and antioxidant defenses, as well as inactivation of JNK, C/EBPδ, and PTEN. Iron loading appeared to induce intermediate stage 1a between stages 1 and 2, as well as stage 2a between stage 2 and AML. At 5 months post-irradiation for stage 1a, 5 mg iron loading in iBMCs (iiBMCs, 5/0) induced alterations that partly resembled stage 2, in which further changes to Akt, NF-κB, JNK, and DNA damage response were observed when compared to stage 1. Conversely, the expression of C/EBPδ and antioxidant genes were altered in stage 2, but this was not the case for the 5 months iiBMCs at stage 1a. Therefore, we concluded that stage 1a is at a more advanced leukemogenic state than stage 1 but has not yet reach stage 2. Moreover, iron loading had additional effects including activation of Foxo3a. The additional effects may be cellular responses to handle the excess iron. Alternatively, non-iron-loaded iBMCs may display a similar expression pattern that resembles stage 1a when they transform from stage 1 to 2. In the 7 months iiBMCs for stage 2a (7.5/0), the tumor suppressor PTEN was downregulated, which is a critical step towards AML development. We therefore concluded that stage 2a is a possible intermediate state between stage 2 and AML. Our ICT regimen did not completely reverse the effects of iron loading on the iron-chelated iiBMCs (ciiBMCs) at 5 (5/40) and 7 (7.5/40) months post-irradiation. ICT partially reduced iron-dependent DNA damage response in the 5 months ciiBMCs, and dampened iron-induced transcription dysregulations of many genes in the 5 and 7 months ciiBMCs, and eventually higher observed AFS. Therefore, we concluded that the leukemogenic states of ciiBMCs were between stage 1 and 1a at 5 months post-irradiation, and between stage 2 and 2a at 7 months post-irradiation.

### A biphasic relationship between iron burden and RI-AML rate

**Fig. 4:**
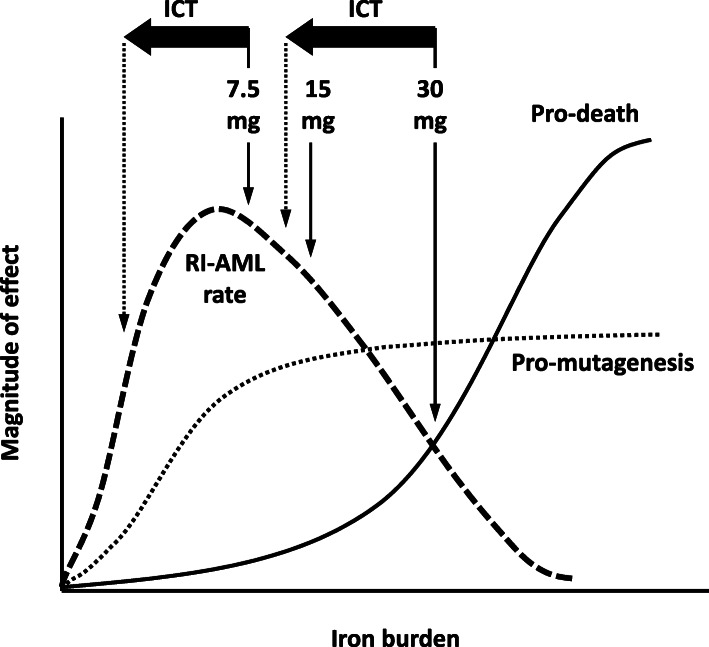
Proposed pro-mutagenic and pro-death influences of iron burden on the rate of RI-AML. The dose-response curve of iron burden with the pro-mutagenesis (dotted line) and pro-death (solid line) responses of HSCs. The RI-AML rate (dashed line) is the combined effect of the pro-mutagenesis and pro-death responses at a given iron dose

We proposed a model to describe the observed RI-AML rate in relation to iron burden (Fig. 4). In this model, iron-induced oxidative stress damages cellular components, including DNA, lipid, and protein [6]. Incomplete repairment of these damages contributes to mutagenesis and subsequently leukemogenesis. The magnitude of the mutagenic effect correlates with iron dose but plateaus after a certain iron burden is reached. Our data on the 7.5 mg iron-loaded mice suggested high pro-mutagenic effect on the HSCs, resulting in peak RI-AML rate. Low-dose ICT decreased the RI-AML rate of the 7.5 mg iron-loaded mice via the apparent reduction of the pro-mutagenic effect (Fig. 2c). On the other hand, iron also has the potential to induce cell death by apoptosis or ferroptosis [7]; induction of cell death by iron has a steeper dose-response curve and surpasses the mutagenic effect at high doses. Thus, cells with exceedingly high iron burden undergo apoptosis or ferroptosis instead of transforming into leukemia-initiating cells. Although we did not perform early analysis with the 15 and 30 mg iron-loaded mice, the lower observed RI-AML rate could be the consequence of increased pro-death effect on the HSCs via iron toxicity, resulting in the decrease of AML-initiating cells. The combined pro-death and pro-mutagenic effects resulted in a biphasic dose-response relationship between RI-AML rate and iron burden. We stratified the RI-AML rate from the lowest at 0 mg iron burden (control group), to 30 mg, 15 mg, and the highest at 7.5 mg – consistent with our observed significant trend in AFS (Fig. 2b, trend *P* < 0.01). Low-dose ICT with the 30 mg iron-loaded mice might have decreased the pro-death effect of iron, but ironically enabled the survival of AML-initiating cells, thereby increased the RI-AML rate (Fig. 2e, control to 30/0 to 30/10, trend *P* < 0.05).

## Discussion

Anemic MDS patients acquire iron overload as a consequence of chronic transfusion [1]. However, unlike thalassemia, the hematopoietic tissues of MDS patients are vulnerable to leukemic transformation. We therefore hypothesized that iron loading may accelerate AML progression in MDS. Our study was designed to investigate the potential of iron loading to induce impairment and eventually AML, specifically when the hematopoietic tissue is in a premalignant state. To answer this question, we used a standard radiation/dexamethasone protocol to induce AML in B6D2F1 mice, which have been utilized commonly in the study of iron loading. Although there has been no report of the rate of RI-AML in B6D2F1 mice, we reasoned that this model should show an intermediate sensitivity to radiation, since it is the F1 progeny of a radiation sensitive (DBA2) and a radiation insensitive (C57Bl/6) strain [4]. Indeed, our B6D2F1 cohort developed RI-AML with prolonged latency at beyond 18 months. For comparison, the frequency of RI-AML in the SJL/J strain is 50–70% at a mean latent period of 10 months after 300 rad irradiation with corticosteroid treatment [4]. Molecular analysis of the iBMCs from the 5 and 7 months cohort suggested progressive gene expression dysregulation consistent with leukemogenesis (Fig. 3), including increased cell survival/proliferation (Akt, NF-κB and Wnt) and decreased apoptosis/tumor suppression (JNK, C/EBPδ and PTEN).

At 7.5 mg, iron was a potent promoter of RI-AML with shortened latency at 6 months and 38% incidence rate. Analysis of the early cohort suggested further gene expression dysregulations in the iiBMCs when compared to iBMCs, which is consistent with the notion of a more advanced pre-leukemic stage for the iiBMCs. The amount of iron burden in this study is generally consistent with the literature and reasonably translatable to the clinical setting. First, a large portion of the reported studies on iron-dependent carcinogenesis were conducted using iron dextran at approximately 21 g [8]. Second, on a per weight basis, 7.5 mg of iron in a mouse is roughly comparable to 60 units of packed RBC for humans – an amount attainable by transfusion dependent MDS patients within the course of their disease [9]. Nevertheless, the iron status of an individual is also influenced by other factors including age, gender, cell/tissue type, and across species [10]. These factors may influence the tolerance and physiological response of BMCs to excess iron, thereby modulating the effects of iron loading to induce cell damage and promote leukemogenesis. Therefore, comparisons of iron sensitivity across studies should be conducted with caution. Moreover, treatments with high-dose deferasirox at 40 mg/kg/day did not appear to be beneficial when compared to their low-dose counterparts. Interestingly, the 30 mg iron-loaded mice acquired other types of tumors instead of AML upon receiving high-dose ICT. The study cohort only received ICT for 8 weeks, and hence a substantial amount of iron remained unchelated. Renal and hepatic toxicity are possible side-effects of deferasirox [11]. Off target effects of deferasirox have also been reported, including NF-kB inhibition [12] and mitochondrial swelling [13]. These unintended effects might have become more apparent with high-dose short-duration ICT, resulting in the shift in oncogenic profile.

We observed a biphasic relationship between iron burden and RI-AML rate, possibly as the combined pro-mutagenic and pro-death effects of iron. A similar biphasic model has been proposed for radiation oncogenesis [14] and demonstrated by Di Majo et al. using irradiated male CBA/Cne mice, in which RI-AML peaked at 3 Gy and declined at 5 and 7 Gy [15]. Radiation-induced BM injury is a valid approach to mimic the pre-leukemic state that includes MDS. Our study confirmed the ability of iron to promote RI-AML. This supports the notion that secondary iron overload as a consequence of chronic transfusion may accelerate AML transformation in MDS. However, the relationship may be complicated due to the biphasic dose-response nature between iron and AML. Risk estimation will require the assessment of the pro-death and pro-mutagenic effects of iron on the HSCs from MDS patients with varying iron burden. Current guidelines regarding the use of ICT in MDS patients with iron overload do not take into account the potential AML risk due to iron overload [16]. It is also uncertain if current recommended serum ferritin target (usually at 1000 μg/L) for ICT is sufficiently low to avoid the potential iron-related AML risk [17]. Thus, our findings point to the necessity of further research to verify the role of secondary iron overload to accelerate AML transformation in MDS.

There were several limitations in our study. We screened a wide range of iron burden by means of iron loading and chelation dosage, which enable the establishment of the relationship between secondary iron overload and RI-AML. Conversely, the statistical power of our findings is limited by the relatively small sample size per treatment group. Jin et al. reported impaired hematopoietic progenitors and shortened overall survival upon iron loading in a MDS mouse model that utilize the *RUNX1-S291fs* mutation [18]. However, the background strain of their model, C57BL/6, is known to be more resistant to both RI-AML development and iron overload [4, 19]. In future study, it would be useful to utilize the genetic variations of iron tolerance between different mouse strains to evaluate the relationship between iron, MDS and leukemogenesis. In addition, the use of total BMCs enabled the assessment of gene expression dysregulation at the population level. Nevertheless, the composition of BMCs, especially at a non-leukemic state, is heterogeneous with different hematopoietic linages at various stages of differentiation. The observed transcriptional changes in this study could also be interpreted as the change in sub-population composition at different time intervals in relation to iron burden. Future investigation can focus on the early hematopoietic progenitors to elucidate the leukemogenic effects of iron on the stem cell niche.

## Conclusions

In this proof-of-concept study, we have demonstrated the ability of iron to promote RI-AML. We also observed a biphasic relationship between iron burden and the rate of RI-AML. ICT reduced the risk of RI-AML at low iron burden, but the effect was reversed at high iron burden. Our findings may have clinical implications to MDS patients who have secondary iron overload due to chronic RBC transfusion.

## Supplementary Information

**Additional file 1.**

## Data Availability

The datasets used and/or analysed during the current study are available from the corresponding author on reasonable request.

## Abbreviations

AFS: AML-free survival
BMCs: Bone marrow cells
iBMCs: Irradiated BMCs
iiBMCs: Iron-loaded iBMCs
ciiBMCs: Iron-chelated iiBMCs
ICT: Iron chelation therapy
MDS: Myelodysplastic syndrome
PB: Peripheral blood
RBC: Red blood cell
RI-AML: Radiation-induced acute myeloid leukemia
ROS: Reactive oxygen species

## References

[1] Leitch HA, Gattermann N (2019). Hematologic improvement with iron chelation therapy in myelodysplastic syndromes: clinical data, potential mechanisms, and outstanding questions. Crit Rev Oncol Hematol..

[2] Remacha AF, Arrizabalaga B, Villegas A, Duran MS, Hermosin L, de Paz R (2015). Evolution of iron overload in patients with low-risk myelodysplastic syndrome: iron chelation therapy and organ complications. Ann Hematol.

[3] Forciniti S, Greco L, Grizzi F, Malesci A, Laghi L. Iron Metabolism in Cancer Progression. Int J Mol Sci. 2020;21:2257. 10.3390/ijms21062257 PMC7139548 32214052

[4] Rivina L, Davoren M, Schiestl RH (2014). Radiation-induced myeloid leukemia in murine models. Hum Genomics.

[5] Kogan SC, Ward JM, Anver MR, Berman JJ, Brayton C, Cardiff RD, Carter JS, de Coronado S, Downing JR, Fredrickson TN, Haines DC, Harris AW, Harris NL, Hiai H, Jaffe ES, MacLennan I, Pandolfi PP, Pattengale PK, Perkins AS, Simpson RM, Tuttle MS, Wong JF, Morse HC, Hematopathology subcommittee of the Mouse Models of Human Cancers Consortium (2002). Bethesda proposals for classification of nonlymphoid hematopoietic neoplasms in mice. Blood..

[6] Gattermann N, Rachmilewitz EA (2011). Iron overload in MDS-pathophysiology, diagnosis, and complications. Ann Hematol.

[7] Sumneang N, Siri-Angkul N, Kumfu S, Chattipakorn SC, Chattipakorn N (2020). The effects of iron overload on mitochondrial function, mitochondrial dynamics, and ferroptosis in cardiomyocytes. Arch Biochem Biophys.

[8] Beguin Y, Aapro M, Ludwig H, Mizzen L, Osterborg A (2014). Epidemiological and nonclinical studies investigating effects of iron in carcinogenesis--a critical review. Crit Rev Oncol Hematol.

[9] Wood EM, McQuilten ZK (2020). Outpatient transfusions for myelodysplastic syndromes. Hematology Am Soc Hematol Educ Program.

[10] Hahn P, Song Y, Ying GS, He X, Beard J, Dunaief JL (2009). Age-dependent and gender-specific changes in mouse tissue iron by strain. Exp Gerontol.

[11] Cappellini MD, Cohen A, Piga A, Bejaoui M, Perrotta S, Agaoglu L, Aydinok Y, Kattamis A, Kilinc Y, Porter J, Capra M, Galanello R, Fattoum S, Drelichman G, Magnano C, Verissimo M, Athanassiou-Metaxa M, Giardina P, Kourakli-Symeonidis A, Janka-Schaub G, Coates T, Vermylen C, Olivieri N, Thuret I, Opitz H, Ressayre-Djaffer C, Marks P, Alberti D (2006). A phase 3 study of deferasirox (ICL670), a once-daily oral iron chelator, in patients with beta-thalassemia. Blood.

[12] Messa E, Carturan S, Maffe C, Pautasso M, Bracco E, Roetto A, Messa F, Arruga F, Defilippi I, Rosso V, Zanone C, Rotolo A, Greco E, Pellegrino RM, Alberti D, Saglio G, Cilloni D (2010). Deferasirox is a powerful NF-kappaB inhibitor in myelodysplastic cells and in leukemia cell lines acting independently from cell iron deprivation by chelation and reactive oxygen species scavenging. Haematologica.

[13] Gottwald EM, Schuh CD, Drucker P, Haenni D, Pearson A, Ghazi S (2020). The iron chelator Deferasirox causes severe mitochondrial swelling without depolarization due to a specific effect on inner membrane permeability. Sci Rep.

[14] Hall EJ (2000). Radiation, the two-edged sword: cancer risks at high and low doses. Cancer J.

[15] Di Majo V, Coppola M, Rebessi S, Saran A, Pazzaglia S, Pariset L (1996). The influence of sex on life shortening and tumor induction in CBA/Cne mice exposed to X rays or fission neutrons. Radiat Res.

[16] Leitch HA (2011). Controversies surrounding iron chelation therapy for MDS. Blood Rev.

[17] Pileggi C, Di Sanzo M, Mascaro V, Marafioti MG, Costanzo FS, Pavia M (2017). Role of serum ferritin level on overall survival in patients with myelodysplastic syndromes: results of a meta-analysis of observational studies. PLoS One.

[18] Jin X, He X, Cao X, Xu P, Xing Y, Sui S, Wang L, Meng J, Lu W, Cui R, Ni H, Zhao M (2018). Iron overload impairs normal hematopoietic stem and progenitor cells through reactive oxygen species and shortens survival in myelodysplastic syndrome mice. Haematologica..

[19] Fleming RE, Holden CC, Tomatsu S, Waheed A, Brunt EM, Britton RS, Bacon BR, Roopenian DC, Sly WS (2001). Mouse strain differences determine severity of iron accumulation in Hfe knockout model of hereditary hemochromatosis. Proc Natl Acad Sci U S A.

