# Supplementary material for: The effects of secondary iron overload and iron chelation on a radiation-induced acute myeloid leukemia mouse model
Source: BMC Cancer. 2021 May 6;21:509. doi: 10.1186/s12885-021-08259-9 (PMC8103632; doi:10.1186/s12885-021-08259-9)
Supplement: Supplementary file 1 — Additional file 1. [file 12885_2021_8259_MOESM1_ESM.docx]

## Supplementary Methods

### Flow cytometry

The BMCs were analyzed by flow cytometry to distinguish different cell populations. All antibodies were purchased from BD Biosciences or eBioscience (San Diego, CA, USA) unless stated otherwise. Flow cytometry was performed with a FACSCalibur cytometer (BD Biosciences, Mississauga, ON, Canada). All data were acquired with Cell Quest software (BD Biosciences) and analyzed with FlowJo for Mac (Tree Star, Ashland, OR, USA). Between 0.5 to 1x10^6^ cells were used per staining set.

The BMCs were stained with a panel of antibodies using standard staining techniques: anti-CD45-APC versus lineage markers (biotin conjugated anti-CD3e, anti-B220, TER119, anti-CD11b, and anti-Gr-1 attached to FITC conjugated streptavidin) (1, 2); myeloid lineage (anti-CD11b-APC) versus lymphoid lineage (biotin conjugated anti-CD3e and anti-B220 attached to FITC conjugated streptavidin); myeloid cell profile (anti-CD11b-APC and anti-Gr-1-FITC) (3); erythroid progenitor profile (anti-CD71-FITC and biotin conjugated TER119 attached to APC conjugated streptavidin) (4).

Intracellular ROS (iROS) level was determined using dichloro-dihydro-fluorescein diacetate (DCFH-DA) (5). BMCs were washed with PBS and resuspended in serum-free RPMI 1640 media (Life Technologies). DCFH-DA was added to the BMCs for a final concentration of 10 µM and incubated at 37^o^C for 15 minutes in a humidified atmosphere of 5% CO_2_ in air. The cells were then washed with PBS, stained by anti-CD45-APC antibody at room temperature for 15 minutes in the dark and analyzed by flow cytometry.

To detect intracellular antigens, BMCs were stained with FITC or APC conjugated anti-CD45 antibodies. The cells were then fixed and permeabilized using the IntraPrep permeabilization reagent (Beckman Coulter, Mississauga, ON, Canada) according to manufacturer’s recommendations. For intracellular staining, the following antibodies were used: Alexa Fluor 647 conjugated anti-phospho-Akt (pAkt) (Ser473) (Cell Signaling Technology, Deverly, MA, USA); anti-phospho-Foxo3a (pFoxo3a) (Ser318/321) (Cell Signaling Technology) with PerCP-Cy5.5 conjugated secondary antibody (Santa Cruz Biotechnology, Dallas, TX, USA); and FITC conjugated anti-phospho-Histone H2A.X (γH2AX) (Ser139) (Millipore Canada). Anti-CD45-FITC stained BMCs were used for pAkt and pFoxo3a staining. Anti-CD45-APC stained BMCs were used for and γH2AX staining. The cells were incubated overnight at 4^o^C and analyzed by flow cytometry. Expression of intracellular antigens was measured in CD45^+^ BMCs.

### DNA, RNA and protein isolation

The remaining BMCs were cryopreserved in freezing medium consisted of 10% dimethyl sulfoxide (DMSO) and 90% FBS. DNA, RNA and protein were isolated from the cryopreserved BMCs at a later time using the AllPrep DNA/RNA/Protein mini kit (QIAGEN, Toronto, ON, Canada) according to manufacturer’s instructions. Their quantity and purity were measured by a NanoDrop 2000c spectrophotometer (Thermo Fisher Scientific, Waltham, MA, USA) using standard methods. All specimens were stored at -70^o^C until further use.

### Oxidative DNA damage (AP sites)

Apurinic/apyrimidinic (AP) site was measured from the genomic DNA of the BMCs using the OxiSelect oxidative DNA damage quantitation kit (Cell Biolabs, San Diego, CA, USA) according to manufacturer’s recommendations.

### Quantitative RT-PCR

The RT^2^ first strand kit (QIAGEN) was used to eliminate genomic DNA contamination and reverse transcribe 400 ng of total RNA into cDNA. The cDNA was then mixed with RT^2^ SYBR Green qPCR mastermix (QIAGEN) and aliquoted into a single RT^2^ Profiler PCR Array (QIAGEN). The following PCR arrays were used: mouse PI3K-Akt signaling pathway (PAMM-058Z), mouse Wnt signaling pathway (PAMM-243Z), and mouse oxidative stress (PAMM-065Z). Each array assesses the expression of 84 genes related to the pathway in question, along with corresponding housekeeping genes and controls. Real-time quantitative polymerase chain reaction (PCR) was performed by a ViiA 7 real-time PCR system (Life Technologies) using recommended settings supplied by QIAGEN. The ΔC_T_ value was calculated by subtracting the C_T_ value of the gene of interest with that of the housekeeping gene (GAPDH). The relative expression value was presented as 2^-ΔC_T_.

### Supplementary Results

### Gene expression in the BMCs of mice that develop AML

We assessed the gene expression profile of BMCs from 5 irradiated mice that developed AML by quantitative RT-PCR using RT^2^ profiler PCR arrays, with which we examined the transcription of 252 genes related to PI3K-Akt, Wnt and oxidative stress. The symbol and description of genes with significantly altered expression are listed in Table S1. In addition to the early cohort (T1.X to T6.X), BMCs from the following mice from the AML group were analyzed: C2.5 (0/0, control mice that developed AML after the 70 weeks observation period), C6.1 (15/0), C7.4 (30/0), C11.4 (7.5/40), and C13.4 (30/10).

A clustergram based on 86 genes was constructed using unsupervised hierarchical clustering analysis and consisted of mice from the AML, 5 and 7 months groups (T1.X to T6.X, Figure S6). As expected, all AML specimens were clustered into the same group, suggesting comparable gene expression profile among the AML mice. Interestingly, one of the iron-loaded mice in the 7 months group (T5.5, 7.5/0) also clustered with the AML mice, suggesting that the BMCs of this mouse could be in a pre-leukemic state. The list of 86 genes comprised 73 genes whose expression levels were altered in the AML group when compared to the 5 months control group based on Student’s t-test, and 13 other genes whose expression levels were altered from other comparisons among the 5 and 7 months groups. For this list of 73 genes (Table S2), 21 were upregulated and 6 were downregulated for genes related to the PI3K-Akt pathway, 5 were upregulated and 16 were downregulated for genes related to the Wnt pathway, and 19 were upregulated and 6 were downregulated for genes related to oxidative stress.

We also compared the AML group with the control groups at different time points to determine if the expression of certain genes is progressively changed and may be involved in leukemogenesis (Table S3). Cebpd, Jun and Nfkbia were progressively downregulated in the irradiated control mice from 5 (T1.X, 0/0) to 7 (T4.X, 0/0) months, and then reached the lowest level for the AML group. Conversely, Fzd7, Itgb1, Ccs and Gss were progressively upregulated from 5 months to 7 months control groups, and then reached the highest level for AML group. We have also examined the expression level of the tumor suppressor phosphatase and tensin homolog (Pten). The mean expression level of Pten in the AML group was not significantly different from the 7 months iron-loaded group. However, we noticed an outlier in the AML group with unusually high level of Pten. Nonetheless, Pten can be regulated by post-translational means and suppressed without altering its transcription level (6). Upon removal of the outlier (C7.4, 30/0), progressive downregulation of Pten was observed from the 7 months control group to the corresponding iron-loaded group and then the AML group (Figure S7).

### The effects of iron on mice at 5 months after irradiation

Next we assessed the *in vivo* effects of iron at 5 months after irradiation on BMCs from control mice (T1.X, 0/0), 5 mg iron-loaded mice (T2.X, 5/0), and mice receiving a 5 mg iron load with 40 mg/kg/day ICT (T3.X, 5/40) (N=5 per treatment group, Figure S1). There was no early mortality and we did not observe visible appearance or weight difference among the 3 treatment groups; none of the mice developed leukemia. BMCs were collected and analyzed at the molecular level. When compared to the control group, we observed lower iROS levels in CD45^+^ BMCs of the iron-loaded group (ANOVA P<0.0001, Table S4). The iROS level in the iron/ICT group was between that of the control (P<0.01) and iron-loaded group (P<0.05). We determined the number of AP sites in the irradiated bone marrow cells and found that some iron-loaded mice have more AP sites (Bartlett’s test for equal variances P<0.001, Table S4). We also used intracellular flow cytometry to examine the phosphorylation of H2AX to γH2AX in response to DNA damage (Table S4). Quantification of γH2AX is a useful tool to detect low levels of DNA damage. We observed significant increase of γH2AX in CD45^+^ BMCs of the iron-loaded group when compared to the control group (ANOVA P<0.05). In the iron/ICT group, γH2AX level was elevated but the difference compared to controls was no longer statistically significant. We further examined the status of the following signaling pathways in CD45^+^ BMCs by intracellular flow cytometry: activation of Akt by Ser473 phosphorylation and activation of Foxo3a by Ser318/321 dephosphorylation. Compared to the control group, both iron and iron/ICT groups had higher level of activated Akt and Foxo3a (Table S4).

We used quantitative RT-PCR arrays to assess the transcription of genes related to PI3K-Akt, Wnt and oxidative stress in the total BMCs for the 5 months cohort (T1.X to T3.X). To screen for transcription dysregulation, the expression level of a given gene of interest from the iron group was compared to that of the control group by Student’s t-test (Table S5). The iron/ICT group was subsequently included, and the 3 groups were evaluated by ANOVA. Although we observed activation of Akt by Ser473 phosphorylation, some PI3K-Akt related genes were downregulated in the iron-loaded mice when compared to the control mice, including Jun (ANOVA P<0.05), Bad (t-test P<0.05), Elk1 (t-test P<0.05), Hras1 (t-test P<0.05), Mtor (t-test P<0.05), and Prkcb (t-test P<0.05). The expression level of Jun was also decreased in the iron/ICT group when compared to the control group (ANOVA P<0.05), while the expression level of other genes was not changed between the control and iron groups. For Wnt related genes, iron-loading was associated with the upregulation of Efnb1 (t-test P<0.01), Fzd7 (ANOVA P<0.01), and Met (ANOVA P<0.01), as well as downregulation of Tcf7l1 (ANOVA P<0.05). In the iron/ICT group, Met remained upregulated (ANOVA P<0.01) and Tcf7l1 remained downregulated (ANOVA P<0.05). For genes related to oxidative stress, Atr was upregulated (t-test P<0.05) in the iron group, while Mpo (ANOVA P<0.05) and Ptgs2 (ANOVA P<0.01) were downregulated. Both Mpo (ANOVA P<0.01) and Ptgs2 (ANOVA P<0.05) remained downregulated in the iron ICT group. There was no significant difference in the expression of Akt1/2 (gene product of Akt) and Foxo3 (gene product of Foxo3a) among the treatment groups.

### The effects of iron on mice at 7 months after irradiation

A second cohort was sacrificed and analyzed after the second iron-loading at 7 months post-irradiation. The cohort comprised mice that were treated with dextran injection (T4.X, 0/0), 7.5 mg iron injection (T5.X, 7.5/0), and 7.5 mg iron injection with 40 mg/kg/day ICT (T6.X, 7.5/40) (N=5 per treatment group, Figure S1). All of the mice from the second cohort were alive with no observable impairment by the time of the analysis. None had developed overt leukemia. BMCs were harvested and analyzed using the same procedure as the 5 months cohort (T1.X to T3.X). We compared the control groups at 5 (T1.X, 0/0) and 7 (T4.X, 0/0) months after irradiation. The level of γH2AX was increased in the 7 months control group (t-test P<0.01) along with activation of Akt (t-test P<0.05) (Table S6). In the PI3K-Akt pathway array, we observed upregulation of Chuk (t-test P<0.05), Ilk (t-test P<0.05), Itgb1 (t-test P<0.05) and Pik3r2 (t-test P<0.01), as well as downregulation of Jun (t-test P<0.05), Nfkbia (t-test P<0.05) (Table S7). In the Wnt pathway array, Cebpd (t-test P<0.05) was downregulated, while Fn1 (t-test P<0.05), Fzd7 (t-test P<0.05), Lrp1 (t-test P<0.05) and Nrp1 (t-test P<0.05) were upregulated (Table S7). Ccs and Gss were upregulated in the oxidative stress array (Table S7). We did not observe significant difference in the expression of Akt1/2 or Foxo3 among the treatment groups.

We then assessed the effects of iron-loading and iron/ICT on mice at 7 months post-irradiation (T4.X to T6.X, Table S8). There were no significant differences for the levels of iROS, γH2AX, pAkt and pFoxo3a among the 3 treatment groups. We also observed comparable expression of genes related to the Wnt pathway and oxidative stress among the 3 groups. On the other hand, iron loading was associated with the downregulation of genes in the PI3K-Akt pathway array, including Grb2 (t-test P<0.05), Itgb1 (t-test P<0.05), Pik3r2 (t-test P<0.05), Pten (ANOVA P<0.05), and Srf (t-test P<0.05). ICT neutralized the effects of iron loading in the irradiated mice in that the expressions of these genes in the ICT group were upregulated when compared to the iron group. Pten expression was higher in the ICT group than the iron group (t-test P<0.05). Moreover, the expression levels of Grb2, Itgb1, Pik3r2 and Srf in the iron/ICT group were not significantly different when compared to the control and iron-loaded groups.

## Supplementary Figures


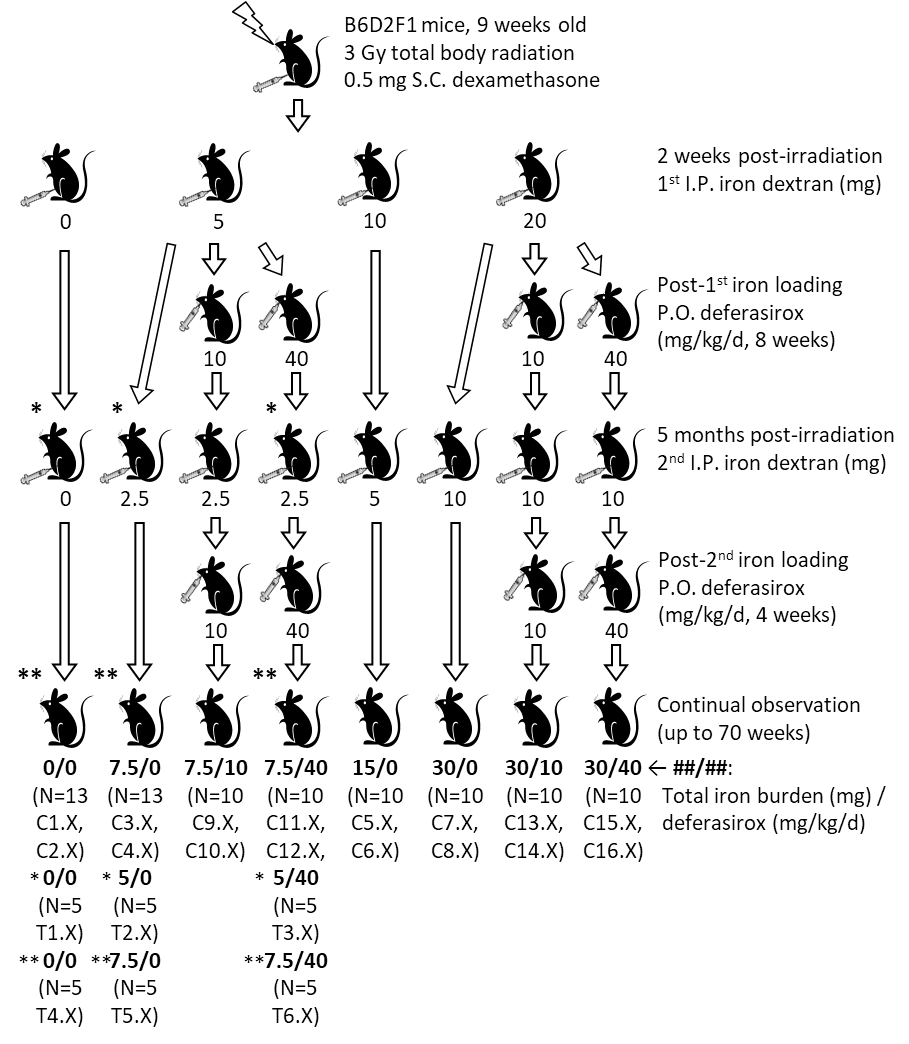


Figure S1. Workflow of iron loading and ICT on irradiated mice. Irradiated mice without additional treatment were labeled C1.X or C2.X. Irradiated iron-loaded mice were labeled C3.X to C8.X. Irradiated iron-loaded mice that also receive ICT were labeled C9.X to C16.X. Early analysis were conducted at 5 (*) and 7 (**) months post-irradiation using 5 mice per treatment. Mice from early analysis at 5 months post-irradiation were labeled T1.X (control, 0/0), T2.X (iron-loaded, 5/0), and T3.X (iron/ICT, 5/40). Mice from early analysis at 7 months post-irradiation were labeled T4.X (control, 0/0), T5.X (iron-loaded, 7.5/0), and T6.X (iron/ICT, 7.5/40).


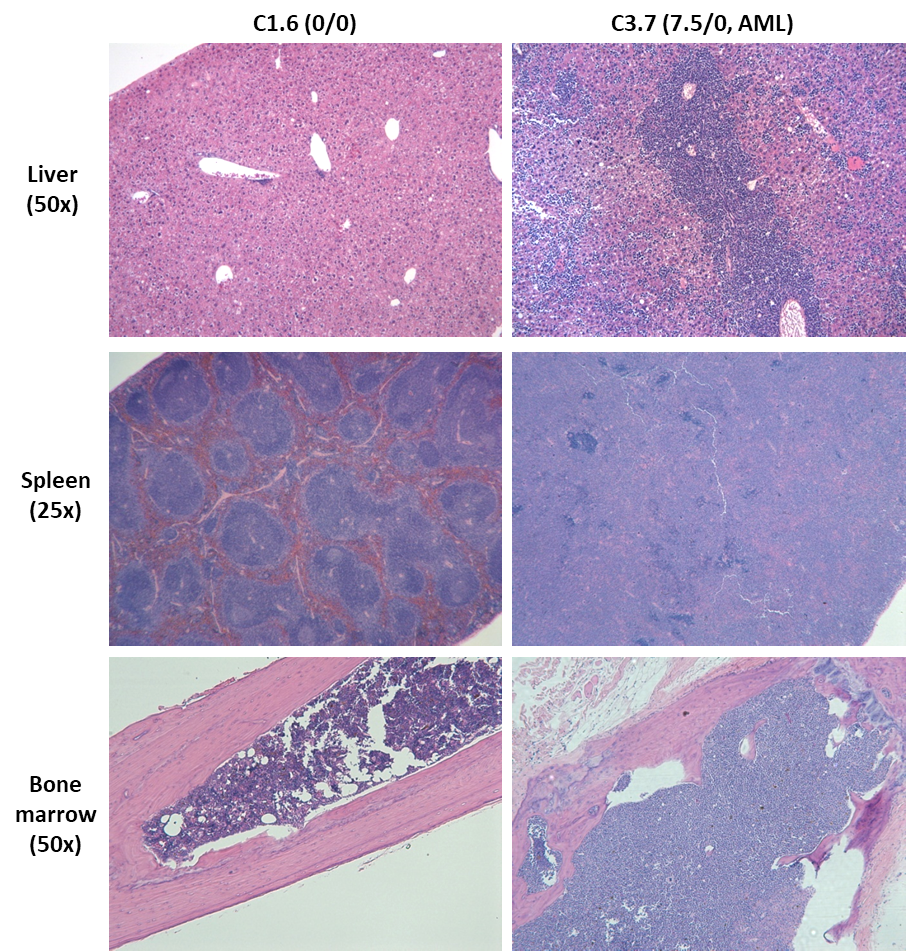


Figure S2. Representative hematoxylin and eosin staining of tissue sections from liver, spleen and bone marrow. Non-iron-loaded C1.6 (0/0) did not display any signs of illness. C3.7 (7.5/0) was loaded with 7.5 mg iron dextran and developed AML. The organs were obtained after the mice were sacrificed. Leukemic blast infiltration was observed in the organs of C3.7.


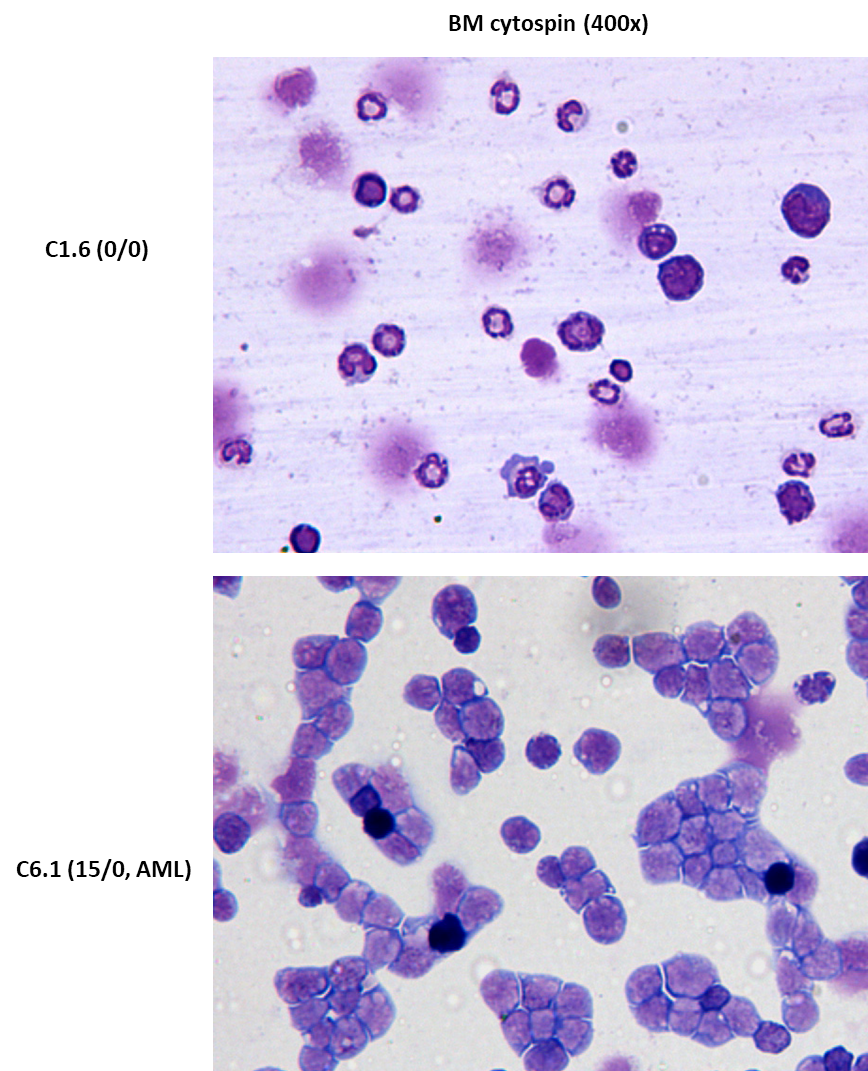


Figure S3. Representative May-Grünwald-Giemsa staining of BM cytospin. Non-iron-loaded C1.6 (0/0) did not display any signs of illness. C6.1 (15/0) was loaded with 15 mg iron dextran and developed AML. The BM specimens were obtained after the mice were sacrificed. The BMCs of C6.1 were more immature and homogenous when compared to that of C1.6.


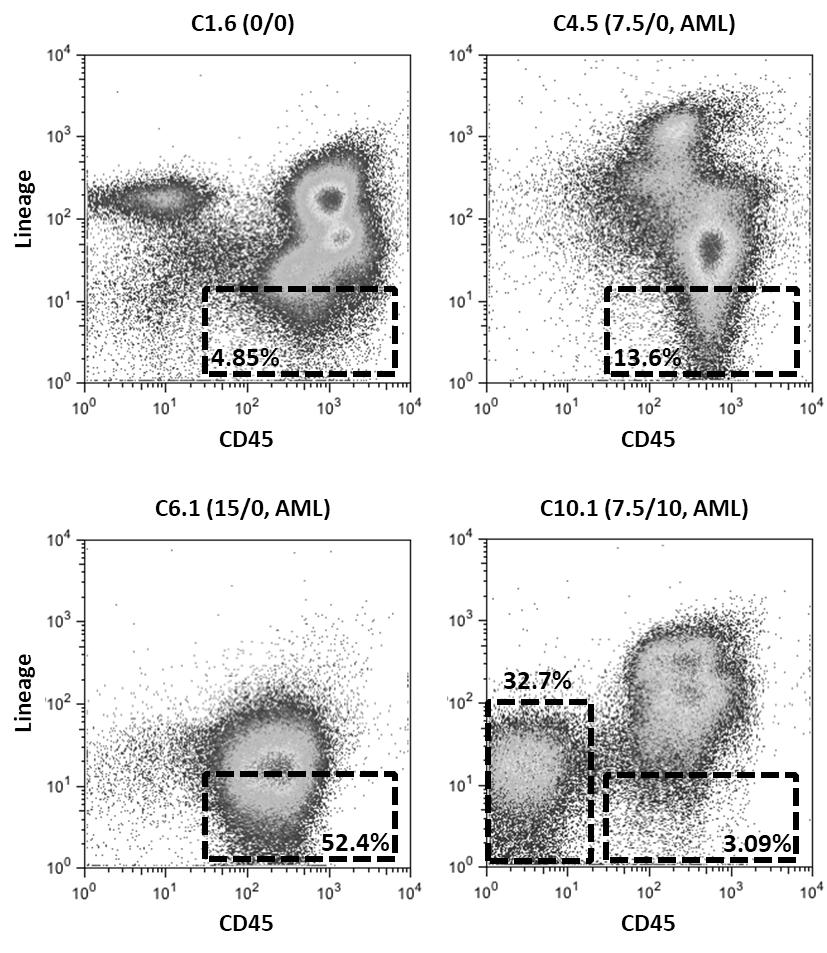


Figure S4. Representative flow cytometry analysis of lineage vs. CD45 BMCs using anti-lineage-FITC and anti-CD45-APC antibodies. The BM specimens were obtained after the mice were sacrificed. Non-iron-loaded C1.6 (0/0) did not display any signs of illness. C4.5 (7.5/0) and C6.1 (15/0) developed monocytic leukemia with increased Lin-CD45+ BMCs. C10.1 (7.5/10) developed monocytic leukemia with increased Lin-CD45low/- BMCs.


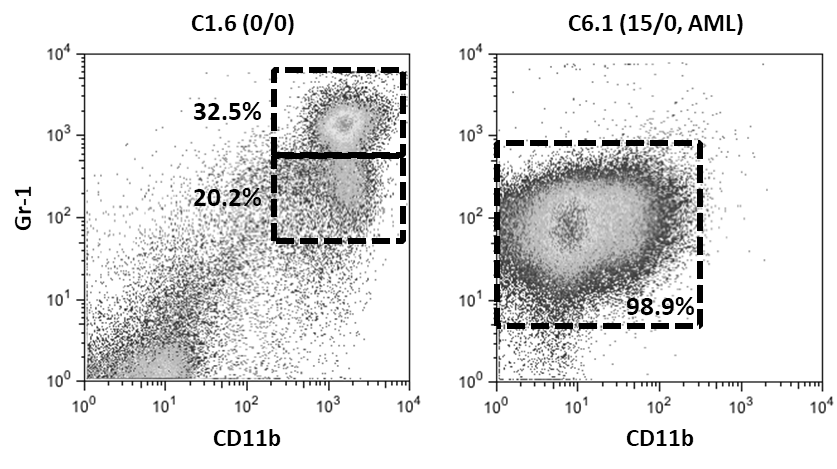


Figure S5. Representative flow cytometry analysis of Gr-1 vs. CD11b BMCs using anti-Gr-1-FITC and anti-CD11b-APC antibodies. The BM specimens were obtained after the mice were sacrificed. Non-iron-loaded C1.6 did not display any signs of illness. C6.1 (15/0) developed monocytic leukemia with CD11b^low/-^Gr-1^+^ BMCs.


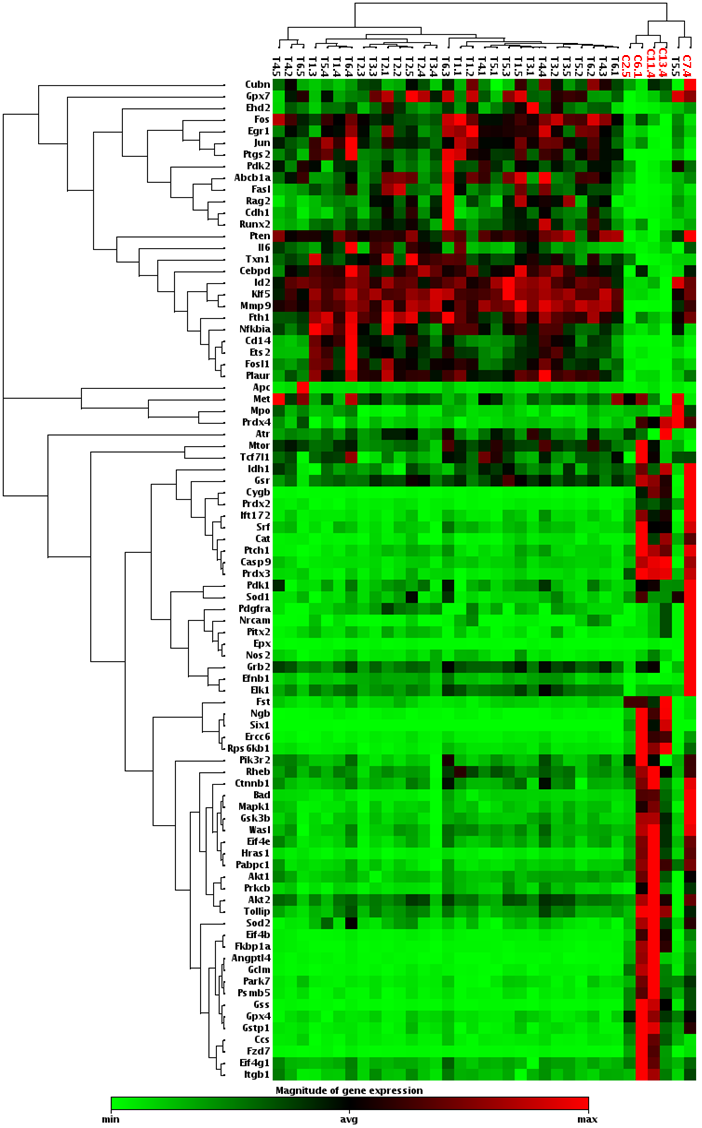


Figure S6. Clustergram of 86 genes that were up or downregulated in the total BMCs of AML mice (C2.5, C6.1, C7.4, C11.4, C13.4 – labeled in red) and all groups used for early analysis (T1.X to T6.X). The clustergram was constructed using 2-dimension, average join type, unsupervised hierarchical clustering analysis.


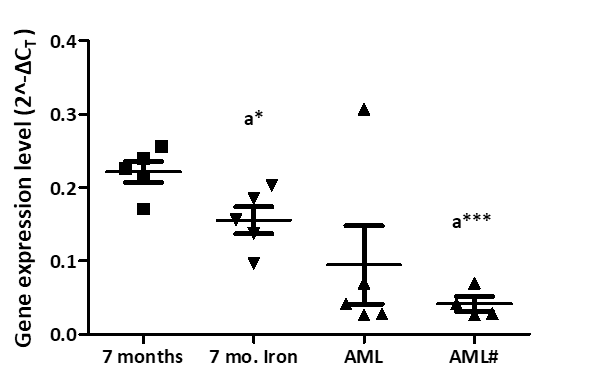


Figure S7. Progressive downregulation of Pten in total BMCs from 7 months irradiated mice (T4.X, 0/0), to 7 months iron-loaded mice (T5.X, 7.5/0), to AML mice. An outlier from the AML group was removed to create the AML# group. Significant differences compared to the 7 months group were marked by “a” for ANOVA. * P<0.05, *** P<0.0001.

## Supplementary Tables

Table S1. Genes with altered expression in all analyses (total BMCs of AML mice, and iBMCs from 5 and 7 months early cohort [T1.X to T4.X]).


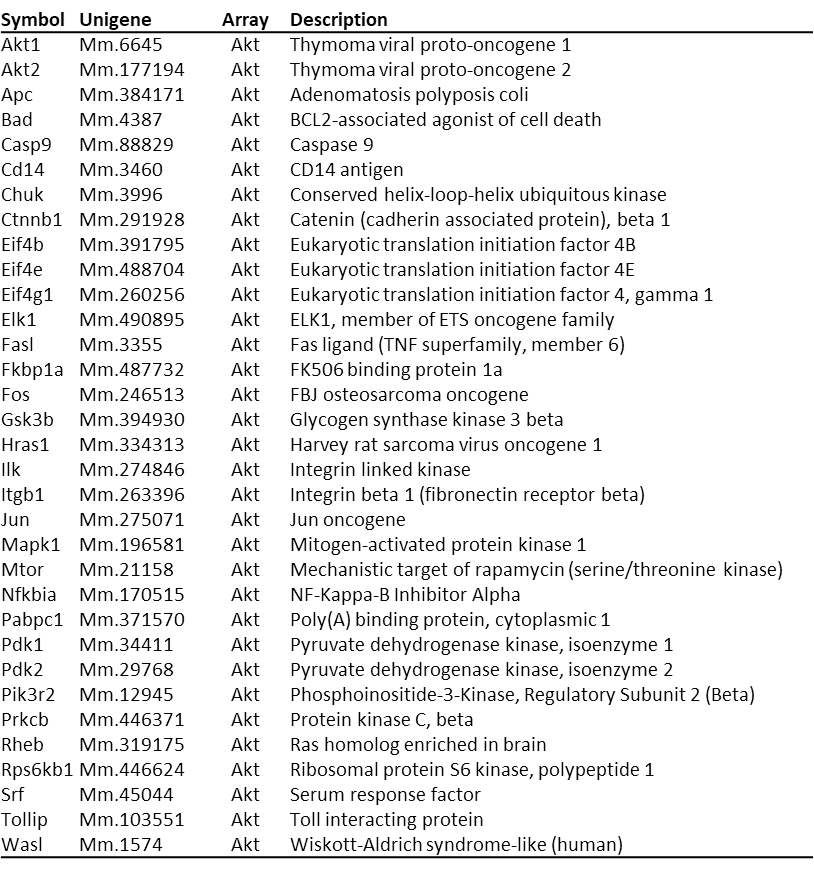


Table S1. Continued (1).


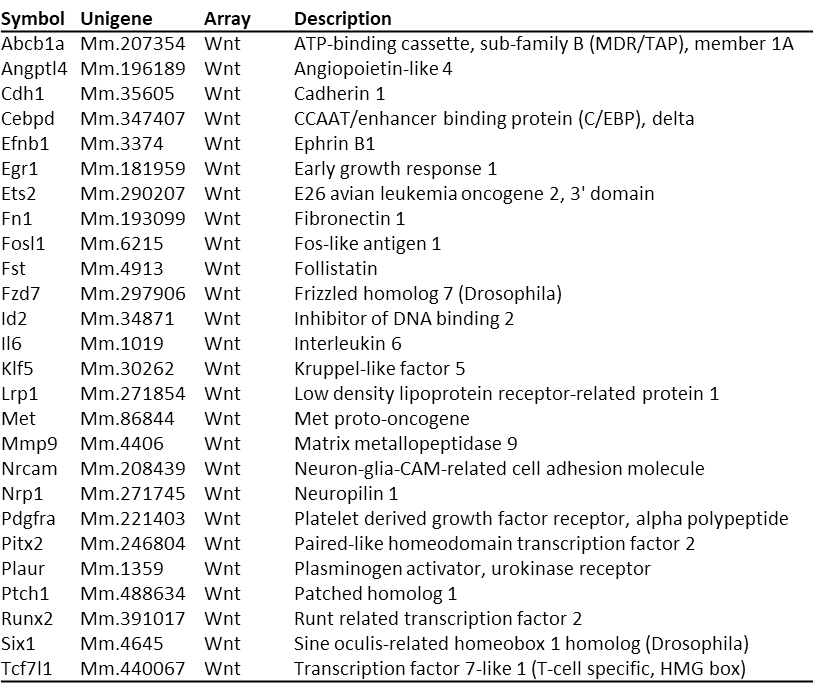


Table S1. Continued (2).


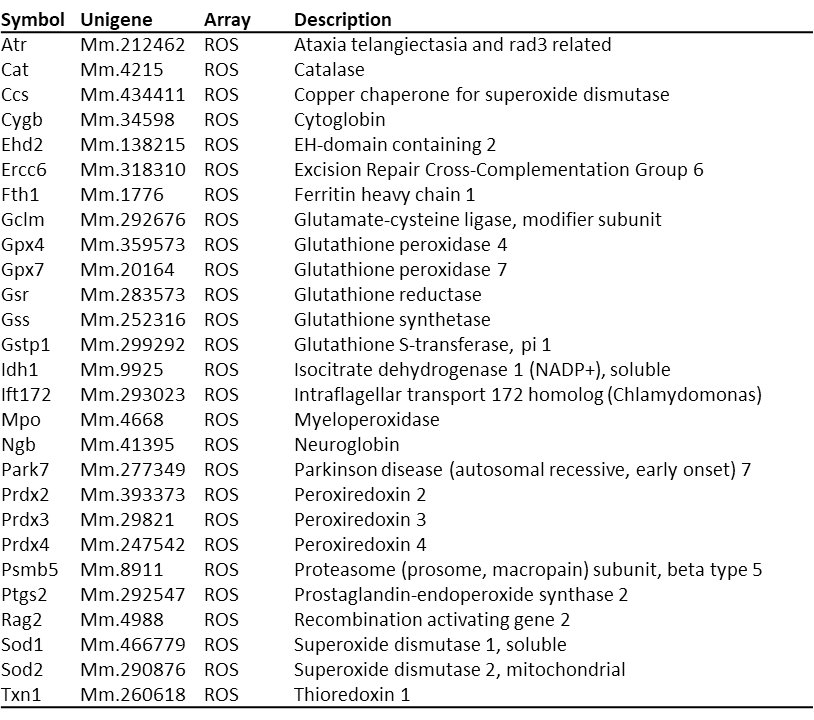


Table S2. Changes in gene expression level of total BMCs (2^-ΔCT value) from 5 months irradiated mice (Control, T1.X,0/0) to AML mice (AML) (t-test P<0.05).


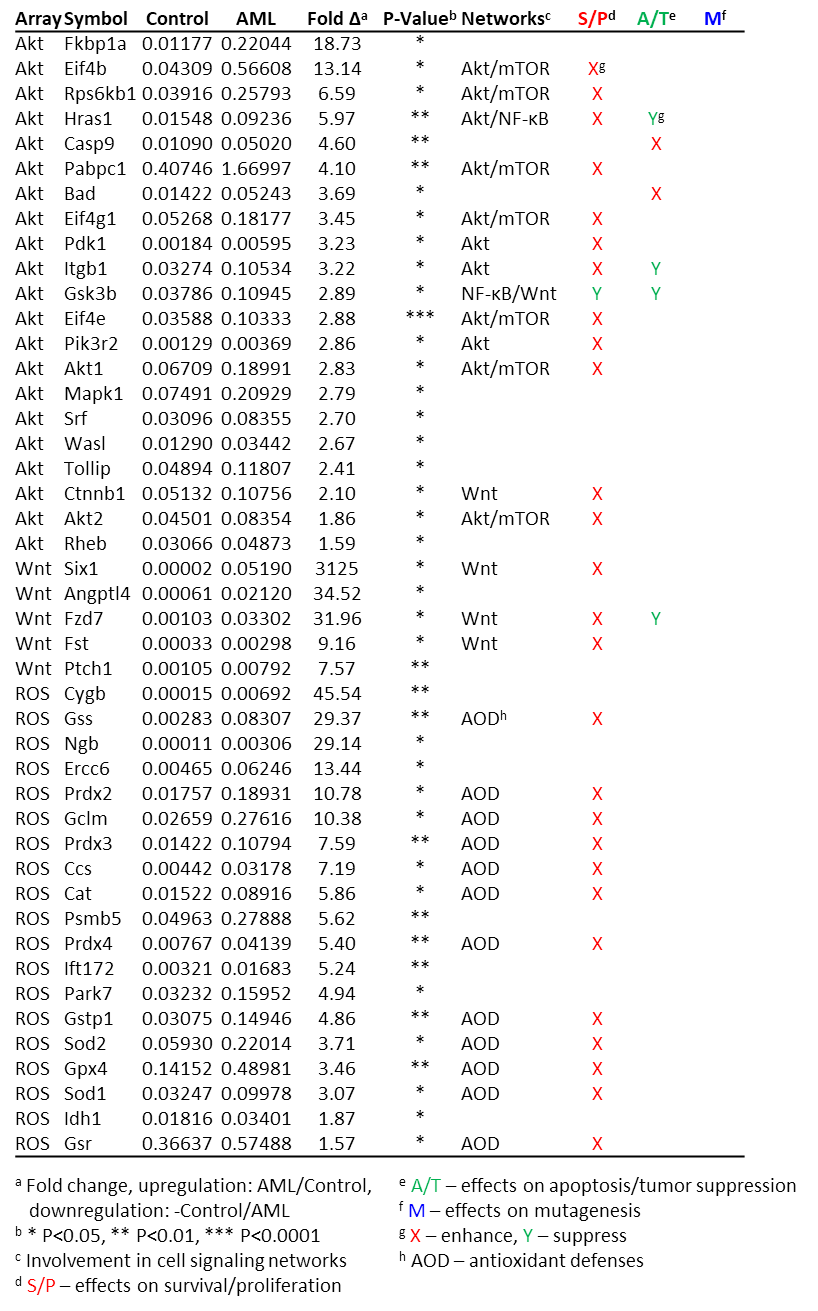


Table S2. Continued.


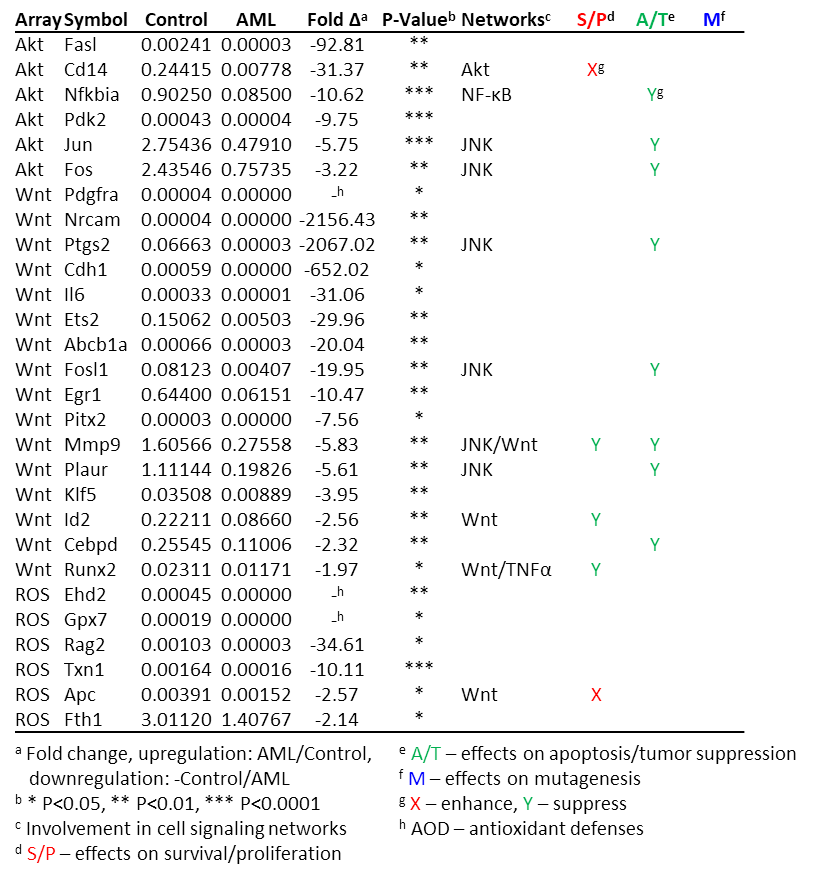


Table S3. Progressive up or downregulation of total BMCs gene expression (2^-ΔCT value) from 5 months irradiated mice (T1.X, 0/0), to 7 months irradiated mice (T4.X, 0/0), to AML mice.


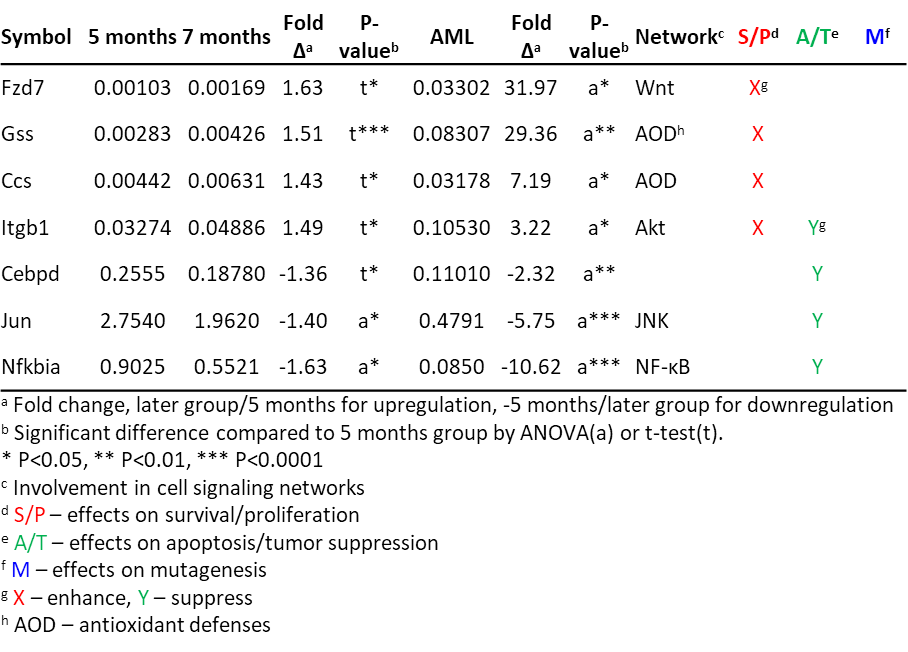


Table S4. Analysis of BMCs from control (T1.X, 0/0, N=5), iron-loaded (T2.X, 5/0, N=5), iron/ICT (T3.X, 5/40, N=5) mice at 5 months post-irradiation. AP sites were measured from the DNA extract of total BMCs. Readings from γH2AX, pAkt, pFoxo3a, and iROS were measured from CD45+ BMCs using flow cytometry.


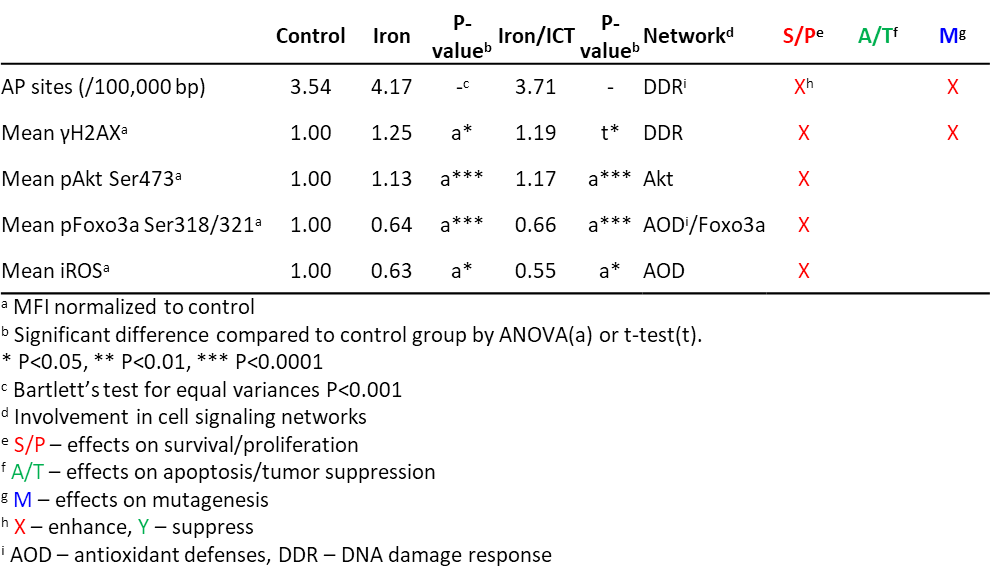


Table S5. Altered gene expression in total BMCs (2^-ΔCT value) from control (T1.X, 0/0), iron (T2.X, 5/0), iron/ICT (T3.X, 5/40) mice at 5 months post-irradiation.


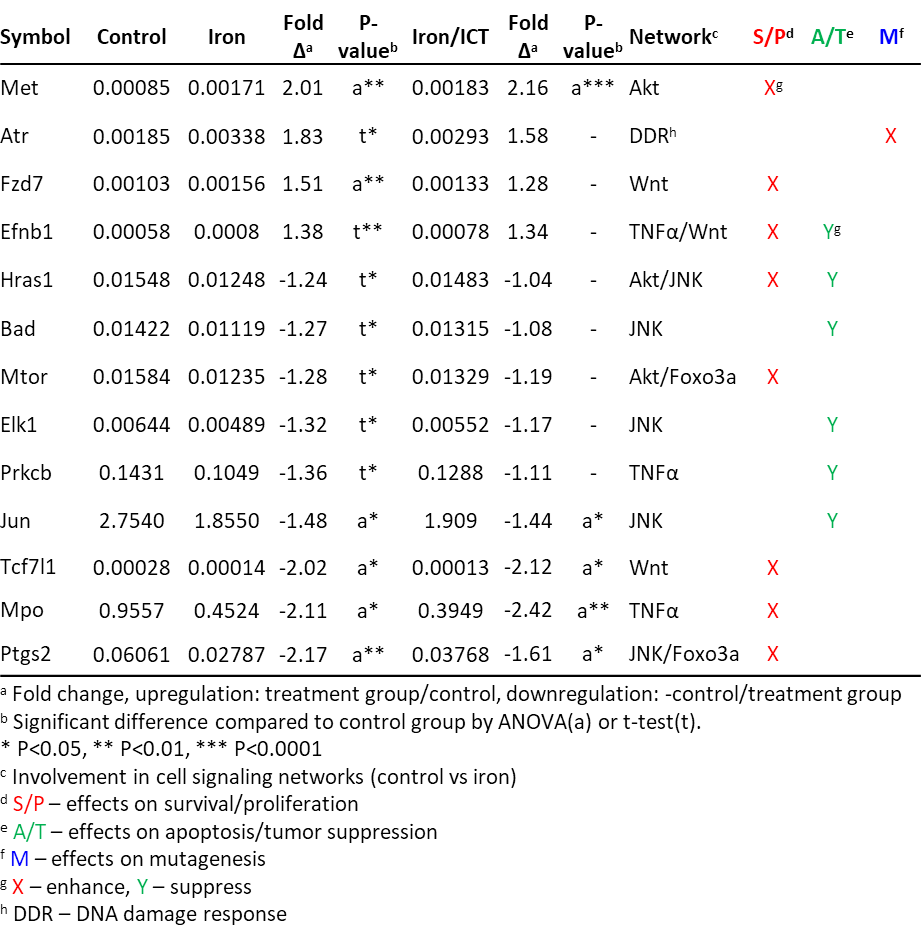


Table S6. Analysis of BMCs from control mice at 5 (T1.X, 0/0) and 7 (T4.X, 0/0) months post-irradiation (N=5 each). Readings from γH2AX, pAkt, pFoxo3a, and iROS were measured from CD45+ BMCs using flow cytometry.


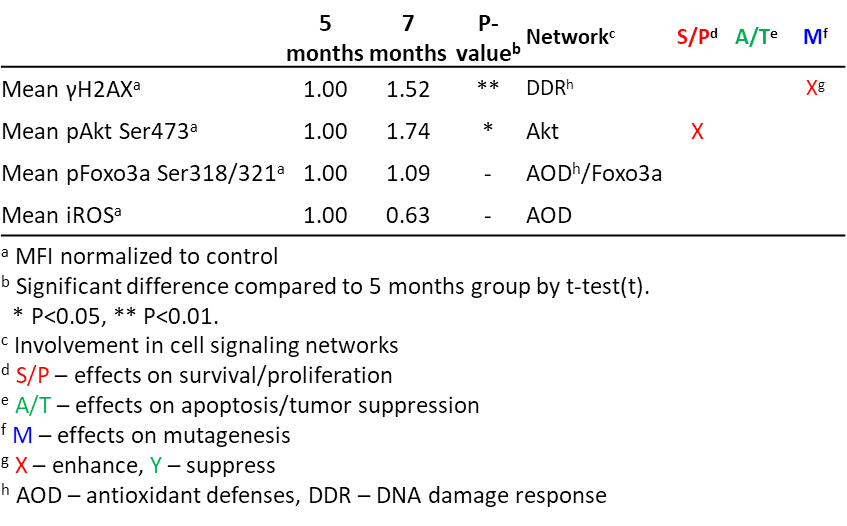


Table S7. Altered gene expression in total BMCs (2^-ΔCT value) from control mice at 5 (T1.X, 0/0) and 7 (T4.X, 0/0) months post-irradiation (N=5 each).


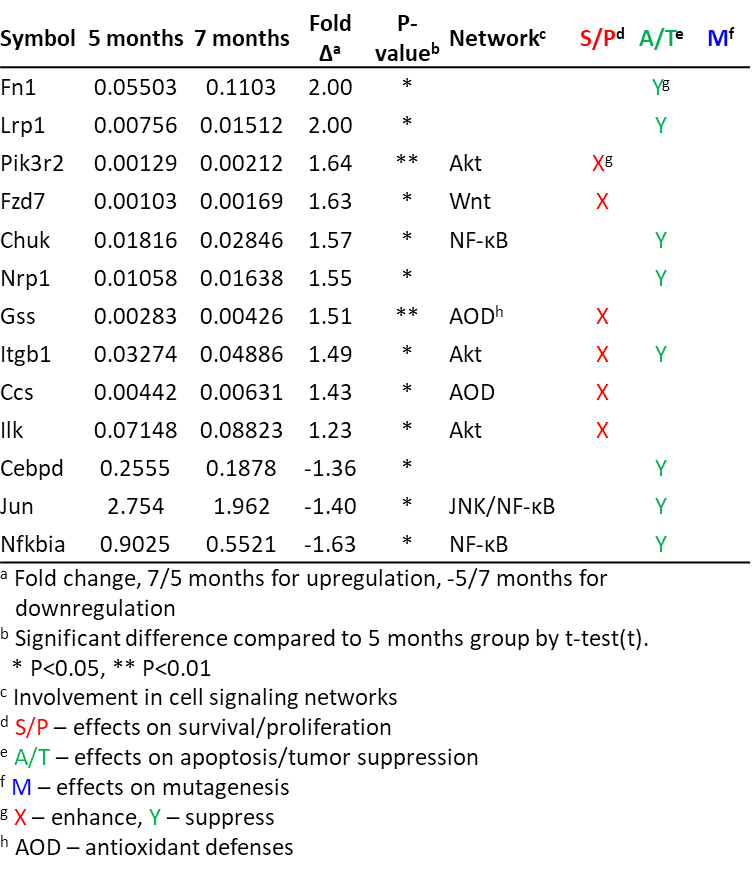


Table S8. Altered gene expression in total BMCs (2^-ΔCT value) from control (T4.X, 0/0), iron (T5.X, 7.5/0), iron/ICT (T6.X, 7.5/40) mice at 7 months post-irradiation.


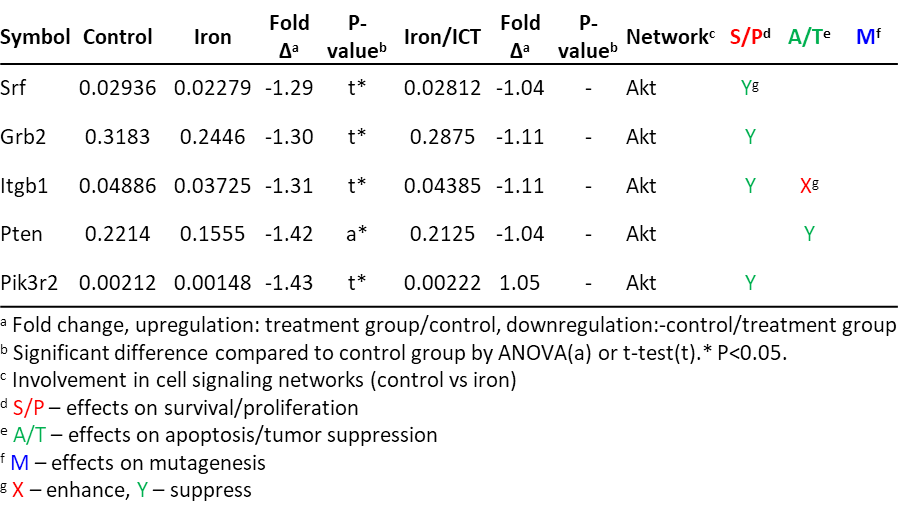


## Supplementary References

1. Stelzer GT, Shults KE, Loken MR. CD45 gating for routine flow cytometric analysis of human bone marrow specimens. Annals of the New York Academy of Sciences. 1993;677:265-80.

2. Weissman IL, Shizuru JA. The origins of the identification and isolation of hematopoietic stem cells, and their capability to induce donor-specific transplantation tolerance and treat autoimmune diseases. Blood. 2008;112(9):3543-53.

3. Lagasse E, Weissman IL. Flow cytometric identification of murine neutrophils and monocytes. J Immunol Methods. 1996;197(1-2):139-50.

4. Koulnis M, Pop R, Porpiglia E, Shearstone JR, Hidalgo D, Socolovsky M. Identification and analysis of mouse erythroid progenitors using the CD71/TER119 flow-cytometric assay. J Vis Exp. 2011(54).

5. Bass DA, Parce JW, Dechatelet LR, Szejda P, Seeds MC, Thomas M. Flow cytometric studies of oxidative product formation by neutrophils: a graded response to membrane stimulation. J Immunol. 1983;130(4):1910-7.

6. Wang X, Jiang X. Post-translational regulation of PTEN. Oncogene. 2008;27(41):5454-63.
